# Hemoglobin-to-Red Cell Distribution Width Ratio and Vitamin D Status as Early Predictors of Cardiovascular Risk in Primary Sjögren’s Syndrome

**DOI:** 10.3390/life16020190

**Published:** 2026-01-23

**Authors:** Francesca Coppi, Francesco Sbarra, Aurora Vicenzi, Cecilia Campani, Martina Moretti, Dilia Giuggioli, Caterina Vacchi, Amelia Spinella, Daniela Aschieri, Anna Vittoria Mattioli, Francesco Fedele, Alessio Baccarani, Marcello Pinti, Alessandra Dei Cas, Federica Fantuzzi, Leila Bigdelu, Gianluca Pagnoni, Susan Darroudi

**Affiliations:** 1 Department of Medical and Surgical Sciences for Children and Adults, University of Modena and Reggio Emilia, Via del Pozzo 71, 41124 Modena, Italy; francesca.coppi@unimore.it; 2 National Institute for Cardiovascular Research (INRC), Via Irnerio 48, 40126 Bologna, Italy; aurora.vicenzi@gmail.com (A.V.);; 3 Cardiology Unit of Emergency Department, Guglielmo da Saliceto Hospital, 29121 Piacenza, Italy; f.sbarra@ausl.pc.it (F.S.);; 4 Cardiology Division, Department of Biomedical Metabolic and Neural Sciences, University of Modena and Reggio Emilia, Via del Pozzo 71, 41124 Modena, Italy; ceci270499@gmai.com (C.C.); moretti.maetina27@gmai.com (M.M.); 5 Rheumatology Unit, Azienda Ospedaliero-Universitaria Policlinico di Modena, University of Modena and Reggio Emilia, 41121 Modena, Italy; dilia.giuggioli@unimore.it (D.G.); vacchi.caterina@outlook.it (C.V.);; 6 Department of Quality-of-Life, University of Bologna—Alma Mater Studiorum, 40126 Bologna, Italy; 7 Emeritus of Cardiology, Sapienza University of Rome, 00185 Rome, Italy; 8 Division of Plastic and Reconstructive Surgery, Department of Medical and Surgical Sciences, Modena Policlinico Hospital, University of Modena and Reggio Emilia, 41124 Modena, Italy; 9 Department of Life Sciences, University of Modena and Reggio Emilia, Via G. Campi 287, 41125 Modena, Italy; 10 Department of Endocrinology and Metabolic Diseases, Parma University Hospital, 43126 Parma, Italy; 11 Department of Medicine and Surgery, University of Parma, 43126 Parma, Italy; 12 Department of Cardiovascular, School of Medicine, Mashhad University of Medical Sciences, Mashhad 91779-48564, Iran

**Keywords:** Sjogren’s disease, hemoglobin-to-RDW ratio, TAPS, TAPSE/PAPs ratio, vitamin D

## Abstract

**Introduction:** Primary Sjögren’s (pSS) is an autoimmune disease that affects several organs, especially the heart, and raises cardiovascular risk. Investigating the associations of hemoglobin-to-red cell distribution width (RDW) ratio (HRR), vitamin D status, and cardiac function could provide valuable insights and biomarkers regarding early cardiovascular risk in patients with pSS. **Method:** This cross-sectional study involved 61 patients diagnosed with pSS based on ACR/EULAR criteria. Data on demographics, hematological (Hb, RDW), echocardiography, and serum vitamin D levels were collected. Echocardiograms were conducted by trained cardiologists following established guidelines, while vitamin D levels were measured using ELISA. Statistical analyses, including univariate linear regression, were performed with SPSS in order to identify whether HRR tertiles were related to cardiac function and vitamin D status. **Results:** A study of 61 pSS patients (mean age 59.8 years, 89% female) revealed that patients with a lower hemoglobin-to-RDW ratio (HRR ≤ 0.98) had significantly higher pulmonary artery pressures (PAPs) and lower values for the tricuspid annular plane systolic excursion (TAPSE)/PAPs ratio, contributing to poor right heart function. These associations were particularly strong in patients with insufficient levels of vitamin D (<30 ng/mL), while differences in other echocardiographic parameters remained nonsignificant between HRR groups. **Conclusions:** These findings underscore the clinical value of HRR as a composite biomarker that reflects the interplay between anemia, inflammation, and cardiovascular health in primary Sjögren’s disease. They also suggest that vitamin D status may be an important therapeutic consideration to mitigate cardiopulmonary risks in this population.

## 1. Introduction

Primary Sjögren’s syndrome (pSS) is a chronic autoimmune disorder characterized primarily by the infiltration of lymphocytes into exocrine glands, leading to dryness of the mucosal surfaces, but it may also involve multiple organ systems beyond the exocrine glands [1]. Although cardiac involvement is not considered a primary manifestation, accumulating evidence suggests that patients with SS are at increased risk of cardiovascular abnormalities, including conduction disturbances, heart failure, and pulmonary hypertension, even in the absence of overt cardiovascular disease [2].

Chronic systemic inflammation, a hallmark of SS, plays a pivotal role in the pathogenesis of cardiovascular disease through endothelial dysfunction, oxidative stress, and microvascular impairment. In this context, hematologic indices, particularly the hemoglobin-to-RDW (HRR), have emerged as potential biomarkers for assessing disease activity and cardiovascular outcomes in various populations, including those with inflammatory and autoimmune diseases [3]. The HRR not only reflects the oxygen-carrying capacity but also serves as a composite measure of inflammation, potentially correlating with cardiovascular disease (CVD) risk [4].

Preliminary evidence indicates that vitamin D deficiency is prevalent among patients with autoimmune disorders, including SS, and has been implicated in the modulation of immune responses and cardiovascular health. The role of adequate vitamin D levels, as immunomodulatory markers, in SS patients was assessed against chronic inflammation and cardiovascular risks, suggesting that vitamin D deficiency may exacerbate the disease’s manifestations and possibly influence HRR and cardiovascular outcomes [2].

In the context of SS, the link between HRR and cardiac function becomes crucial, given that patients with severe manifestations often exhibit elevated inflammatory markers and altered hematologic profiles. Studies have shown that lower levels of HRR are significantly associated with increased risks of mortality and CVD [5]. Moreover, the role of vitamin D status in SS has gained attention due to its immunomodulatory functions, suggesting that vitamin D deficiency may exacerbate the disease’s manifestations and potentially influence HRR and cardiovascular outcomes [5].

Despite emerging evidence linking hematological indices and vitamin D with cardiovascular outcomes in autoimmune diseases, their interplay in the context of SS remains underexplored [3]. Studies have shown that lower levels of HRR are significantly associated with increased risks of mortality and CVD. Furthermore, the significant role of vitamin D in modulating immune responses suggests that vitamin D deficiency may exacerbate the disease’s manifestations and possibly influence HRR and cardiovascular outcomes [6]. Addressing this gap may improve the understanding of the mechanisms underlying subclinical cardiovascular involvement in SS and support the identification of accessible biomarkers for early cardiovascular risk stratification. Therefore, the present study aimed to evaluate the relationship between the hemoglobin-to-RDW ratio and cardiac function in patients with primary Sjögren’s syndrome, with a specific focus on the modifying role of vitamin D status.

## 2. Methods

### 2.1. Study Population and Data Collection

This cross-sectional study included 61 patients diagnosed with primary Sjögren’s syndrome (pSS) at Policlinico di Modena, Modena, Italy. All patients met the 2016 ACR/EULAR classification criteria for Sjögren’s syndrome (pSS) [7]. Data collection took place from November 2022 to April 2024 at the F. Coppi et al. [8] International Journal of Cardiology 430 (2025) 1331852 Cardiology Unit of the University Hospital of Modena and included 2D/3D echocardiography, ECG, clinical and biochemical assessments, HRCT, and pulmonary function tests. The study protocol was approved by the ethics committee at the University of Modena and Reggio Emilia, Italy, on 19 July 2022. Inclusion criteria comprised patients aged 18 years or older with a confirmed diagnosis of Sjögren’s syndrome. Patients had no data of hematology parameters and echocardiographic features were excluded. Baseline demographic, biochemical, and hematological data were systematically recorded for all patients enrolled at Modena Polyclinic and in the laboratory of the Polyclinic. Hemoglobin-to-RDW ratio calculated by dividing Hb by RDW. HRR is categorized based on its tertiles.

### 2.2. Ethics Statement

The study protocol was conducted based on the Helsinki Declaration and was approved by the local ethics committee, Area Vasta Emilia Nord (protocol no. 275/16). Written informed consent was obtained from all study participants [8,9].

### 2.3. Cardiological Evaluation

Cardiovascular risk factors, such as obesity, diabetes, dyslipidemia, smoking, chronic renal failure, and systemic hypertension, were recorded. ECGs were conducted to detect arrhythmias, and 2D/3D echocardiography was assessed with a Philips EPIQ ultrasound by 2 experienced cardiologists in specialized echocardiography. Standardized echocardiographic measurements and interpretations were provided by the American Society of Echocardiography and the European Association of Cardiovascular Imaging. Structural and functional parameters of both right and left heart chambers were assessed [9].

### 2.4. Vitamin D Assessment

Serum 25-hydroxyvitamin D levels were measured at the Clinical Laboratory using an enzyme-linked immunosorbent assay (ELISA), specifically designed for vitamin D quantification. Serum levels were set to <30 ng/mL as vitamin D inadequacies according to general clinical guidelines. Levels above this value were deemed adequate [9].

### 2.5. Statistical Analysis

Statistical analysis was performed using SPSS version 26 (IBM Corporation, Chicago, IL, USA). Data were reported as mean ± SD for quantitative and number (%) qualitative data. Normal distribution was determined by the Kolmogorov–Smirnov test. A chi-square test was used to compare categorical groups, and the Kruskal–Wallis H test was utilized to compare quantitative data between groups. To evaluate the association between echocardiographic parameters as dependent variables and HRR tertiles as independent variables (with T3 as the reference group compared to T1 and T2), β-coefficients were calculated using univariate linear regression models. The data were adjusted for potential confounding factors, including age, sex, smoking status, BMI, DM, HTN, dyslipidemia, ILD, and duration of disease. A *p*-value < 0.05 was considered significant. GraphPad version 8.0.2 was used to create a graph.

## 3. Results

The study involved 61 participants with Sjögren’s disease, stratified according tovit Damin D status, who are summarized in Table 1. A total of 61 patients were included, of whom 23 had sufficient vitamin D levels (≥30 ng/mL) and 38 had vitamin D deficiency (<30 ng/mL). The mean age in vit D ≥ 30 ng/mL group was 58.91 ± 12.98 years and in vit D < 30 ng/mL group was 59.53 ± 15.31. The majority were female (88.9%), and 11.1% were male. Nineteen percent were current smokers, while 38.1% had interstitial lung disease (ILD). The mean body mass index (BMI) was significantly higher in patients with vit D < 30 ng/mL. The prevalence of comorbidities was 32.8% for hypertension, 23.4% for dyslipidemia, and 6.7% for diabetes mellitus; the mean hemoglobin concentration was 13.2 ± 1.66 g/dL, red cell distribution width (RDW) was 14.59 ± 1.73%, and the mean HRR was 00.91 ± 0.18 in patients with vit D < 30 ng/mL (Table 1). There were no significant differences between the two groups in terms of Hb, RDW, and HRR (*p* < 0.05).

Echocardiographic assessments were compared between the two groups. The mean TAPSE was 23.44 ± 3.8 mm, STDI was 12.36 ± 2.4 cm/s, PAPs were 27.58 ± 7.38 mmHg, and a TAPSE/PAPs ratio was 0.92 ± 0.35 in patients with vit D < 30 ng/mL. The mean FAC_RV in patients with vit D ≥ 30 and <30 ng/mL was 39.62 ± 8.19 and 40.81 ± 11.51%, respectively, and EF_RV was 47.27 ± 6.48% in patients with vit D ≥ 30 and 49.01 ± 7.07% in patients with vit D < 30 ng/mL (Table 1).

**Figure 1:**
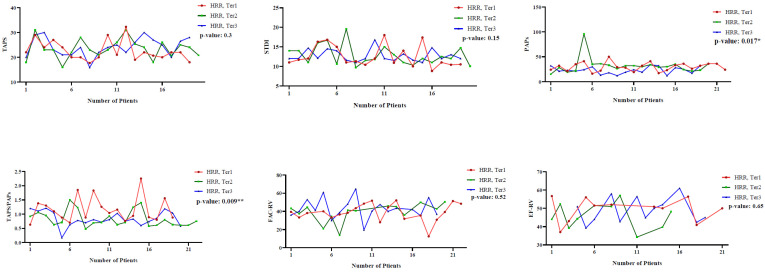
Comparison of echocardiography parameters *p*-value in total patients according to HRR tertiles. HRR tertiles (hemoglobin-to-RDW ratio); T1 < 0.87, T2: 0.87–0.98, and T3 > 0.98; * *p*-value < 0.05, ** *p*-value: 0.01–0.001. TAPSE: tricuspid annular plane systolic excursion, PAP: systolic pulmonary arterial pressure, STDI: tricuspid annular systolic velocity, TAPSE/PAPs: TAPSE to PAPs ratio, FAC: fractional area change, RV: right ventricle, EF: ejection fraction.

The echocardiographic parameters were analyzed according to HRR tertiles (T3 > 0.98, T2: 0.87–0.98, T1 < 0.98) in the total patients with Sjögren’s disease, as well as stratified by vitamin D status (≥30 ng/mL and <30 ng/mL) (Table 2 and Figure 1). In the total patient group (N = 61), PAPs were significantly lower in patients with HRR > 0.98 compared to other groups (22.95 ± 7.02 mmHg vs. 22.95 ± 7.02 and 29.68 ± 8.63 mmHg, *p* < 0.05). Additionally, the TAPSE/PAPs were significantly higher in patients with HRR > 0.98 (*p* < 0.05). All other parameters, including TAPSE, STDI, FAC_RV, and EF_RV, did not differ significantly between the HRR groups (Figure 1).

“Likewise, comparing patients with vitamin D < 30 ng/mL ranked in order of vitamin D levels, this trend matched; PAPs was significantly lower (*p* < 0.05), and the TAPSE/PAPs ratio was significantly higher (*p* < 0.05) in the highest HRR tertile compared to lower tertiles.”. In comparison, PAPs and TAPSE/PAPs were not statistically different for patients with vitamin D ≥ 30 ng/mL between HRR tertiles (*p* > 0.05). No differences were seen in other parameter values in HRR tertiles with respect to each group in which there was a vitamin D subgroup (Table 2).

**Figure 2:**
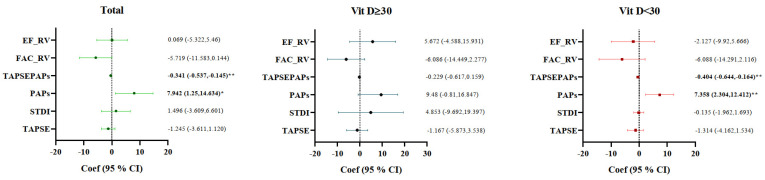
Determining factors associated with echocardiography parameters (dependent variables) in patients with Sjögren’s syndrome using a linear regression model according to HRR tertiles (independent variable); T1 and T2 as one group and compared with T3 > 0.98 (reference). Data adjusted for age, sex, smoking status, BMI, DM, HTN, dyslipidemia, ILD, and duration of disease. * *p*-value < 0.05, ** *p*-value: 0.01–0.001. HRR: hemoglobin-to-RDW ratio, TAPSE: tricuspid annular plane systolic excursion, PAP: systolic pulmonary arterial pressure, STDI: tricuspid annular systolic velocity, TAPSE/PAPs: TAPSE to PAPs ratio, FAC: fractional area change, RV: right ventricle, EF: ejection fraction.

We used linear regression analysis to identify the association between the echocardiographic parameters and HRR tertiles among Sjögren’s disease patients, stratified by vitamin D status. The highest tertiles (T3, HRR > 0.98) were defined as the reference group, and the model was adjusted for age, BMI, smoking, hypertension, diabetes, and dyslipidemia. Patients in the lower HRR tertiles (T1 + T2 ≤ 0.98) had significantly higher PAPs (β = 7.94, 95% CI 1.25–14.63, *p* < 0.05) and lower TAPSE/PAPs ratios (β = −0.341, 95% CI −0.537 to −0.145, *p* < 0.05) when compared with the reference group. PAPs: β = 7.36 (95% CI 2.30–12.41, *p* < 0.05), TAPSE/PAPs: β = −0.404 (95% CI −0.644 to −0.164, *p* < 0.05); these associations remained significant in the subgroup having vitamin D < 30 ng/mL, while no significant effects were observed in patients with vitamin D > 30 ng/mL. In addition, TAPSE, S′ (STDI), FAC, and EF showed no statistically relevant differences in the HRR groups, yet FAC exhibited a trend toward reduction in patients with low HRR (β = −5.72, *p* > 0.05) (Figure 2).

## 4. Discussion

Our findings demonstrate that a lower HRR is associated with adverse echocardiographic markers of right ventricular–pulmonary vascular coupling, particularly increased pulmonary artery pressures, and decreased TAPSE/PAPs ratios in patients with Sjögren’s disease. Notably, these associations were more pronounced in patients with concomitant vitamin D insufficiency, suggesting that vitamin D status may modulate the relationship between hematological indices and cardiopulmonary involvement in this population.

Elevated RDW and altered HRR have previously been implicated as prognostic hematologic indices in various cardiovascular conditions and inflammatory diseases [3,10]. Prior studies have shown that elevated RDW and reduced HRR are associated with increased mortality and adverse cardiovascular outcomes in heart failure, coronary artery disease, and acute coronary syndrome. Compared with hemoglobin or RDW alone, HRR has been proposed as a more robust marker reflecting the combined effects of anemia and inflammation [11,12]. A cohort study indicated that a unit increase in HRR is linked to significant reductions in both 28-day and 90-day hospital mortality rates [5]. However, data examining HRR in autoimmune diseases, particularly in Sjögren’s disease, remain scarce.

Chronic systemic inflammation is a central feature of Sjögren’s syndrome and plays a key role in both hematologic alterations and cardiovascular involvement. It induces a persistent pro-inflammatory state mediated by cytokines (IL-1, IL-6, TNF-α) [13]. These conditions inhibit erythropoietin production and iron metabolism (hepcidin increase), contributing to anemia of chronic disease (lower hemoglobin). Concurrently, oxidative stress and inflammatory damage to the erythrocytes increase red blood cell size heterogeneity, reflected by elevated RDW [14,15]. Studies indicate that Sjögren’s patients with higher systemic inflammation may exhibit elevated RDW [16,17]. Anemia and increased RDW, and consequently low HRR, are frequently observed in SS. This impaired loop may lead to worsened inflammation, oxidative stress, and heart failure [18,19].

Several studies have shown that a reduced hemoglobin-to-RDW ratio (HRR) has been associated with the negative outcomes seen in heart failure, coronary artery disease, and acute coronary syndrome [3,4]. Our finding is consistent with this result and shows an association between a reduced HRR and impaired right ventricular–pulmonary vascular coupling. Moreover, this ratio is associated with higher mortality, increased subsequent rehospitalization, and adverse cardiac events. The underlying mechanisms may involve chronic inflammation that stimulates cytokines (such as IL-6 and TNF-α) and impairs iron metabolism and erythropoiesis. This causes an increased RDW and decreased hemoglobin production [20]. Anemia and erythrocyte dysfunction can compromise oxygen delivery to the myocardial and peripheral tissues, thereby exacerbating hypoxia-induced pulmonary vasoconstriction and increasing pulmonary artery pressures. In parallel, an elevated RDW has been linked to endothelial dysfunction, oxidative stress, and vascular remodeling, all of which contribute to pulmonary hypertension and right ventricular dysfunction. These mechanisms may partially explain the observed relationship between a lower HRR and reduced TAPSE/PAPs ratio, a validated marker of right ventricular–pulmonary arterial coupling and a prognostic indicator in pulmonary vascular disease [21].

Cardiac tissue expressed vitamin D receptors highly, so that vitamin D regulates cardiomyocyte hypertrophy, fibrosis, and myocardial contractility [22]. On the other hand, vitamin D inhibits NF–κB signaling, a central pathway in inflammatory gene activation in SS [23]. Reducing vit D in SS leads to chronic inflammation, endothelial dysfunction, and accelerated atherosclerosis [24,25]. Furthermore, it can exacerbate anemia and alter hematologic indices. This complex increases the risk of hypertension, coronary artery disease, heart failure, and adverse cardiac remodeling. Our findings suggest that vitamin D insufficiency may potentiate inflammation-related hematologic disturbances, resulting in a lower HRR and greater cardiopulmonary impairment. This observation provides novel insight into the potential interaction between vitamin D status, hematologic biomarkers, and subclinical cardiovascular involvement in Sjögren’s disease.

Mechanistically, the chronic inflammation characteristic of Sjögren’s syndrome promotes cytokine-mediated disruptions in erythropoiesis and iron metabolism, leading to anemia and increased red blood cell size heterogeneity [8,26]. This results in a decreased HRR, which correlates with intensified oxidative stress, endothelial dysfunction, and myocardial injury, thereby contributing to adverse cardiac remodeling and heart failure.

The study has several limitations. The small sample size and single center are the most important constraints. A multicenter study would be beneficial in collecting more samples from a diverse range of races and populations. Another concern is the cross-sectional approach, which could be enhanced by longitudinally tracking patients over time and evaluating their vitamin D levels, hematological parameters, and cardiac function.

## 5. Conclusions

This study highlights the clinical significance of the hemoglobin-to-RBC distribution width (HRR) ratio as a comprehensive biomarker in Sjögren’s disease. Taken together, our results support the concept that HRR may serve as a low-cost, readily available biomarker reflecting the combined effects of chronic inflammation, oxidative stress, and impaired erythropoiesis in Sjögren’s syndrome. When considered alongside vitamin D status, the HRR may offer additional value for identifying patients at higher risk of cardiopulmonary involvement before the development of overt cardiovascular disease. Furthermore, the research indicates that vitamin D levels may play a crucial role in managing cardiopulmonary risks within this patient population. This finding proposes designing a prospective and interventional study to confirm the role of vitamin D supplementation and the potential role of the HRR in cardiovascular risk among patients with pSS.

## Figures and Tables

**Table 1:** Baseline data of patients with Sjögren’s syndrome according to vit D.

		Vit D ≥ 30, n: 23	Vit D < 30, n: 38	*p*-Value
Age, y		58.91 ± 12.98	59.53 ± 15.31	>0.05
Sex	Male	0	7 (18.4)	0.038
	Female	23 (100)	31 (81.6)	
Smoking	No	21 (91.3)	28 (73.7)	>0.05
	Yes	2 (8.7)	10 (26.3)	
ILD	No	13 (56.5)	25 (65.8)	>0.05
	Yes	10 (43.5)	13 (34.2)	
BMI		23.57 ± 4.68	26.07 ± 4.6	0.048
Vit D, ng/mL		40.79 ± 10.34	17.45 ± 6.86	<0.001
HTN	No	12 (57.1)	27 (71.1)	>0.05
	Yes	9 (42.9)	11 (28.9)	
Dyslipidemia	No	16 (72.7)	29 (76.3)	>0.05
	Yes	6 (27.3)	9 (23.7)	
Diabetes	No	22 (100)	32 (88.9)	>0.05
	Yes	0	4 (11.1)	
HB, mg/dL		12.72 ± 1.21	13.2 ± 1.66	>0.05
RDW, %		14.44 ± 1.62	14.59 ± 1.73	>0.05
HRR		0.89 ± 0.15	0.91 ± 0.18	>0.05
TAPSE, mm		23.69 ± 4.45	23.44 ± 3.8	>0.05
STDI, cm/s		12.34 ± 2.59	12.36 ± 2.4	>0.05
PAPs, mmHg		29.43 ± 16.86	27.58 ± 7.38	>0.05
TAPSE/PAPs		0.95 ± 0.37	0.92 ± 0.35	>0.05
FAC_RV, %		39.62 ± 8.19	40.81 ± 11.51	>0.05
EF_RV, %		47.27 ± 6.48	49.01 ± 7.07	>0.05
Duration of Sjögren’s disease		6.6 ± 4.98	7.52 ± 7.23	>0.05

Data presented as mean ± SD or number and percentage. BMI: body mass index, HTN: hypertension, HB: hemoglobin, RDW: red blood distribution, HRR: hemoglobin-to-RDW ratio, ILD: interstitial lung disease, TAPSE: tricuspid annular plane systolic excursion, PAP: systolic pulmonary arterial pressure, STDI: tricuspid annular systolic velocity, TAPSE/PAPs: TAPSE to PAPs ratio, FAC: fractional area change, RV: right ventricle, EF: ejection fraction.

**Table 2:** Echocardiography parameters according to HRR tertiles in Sjögren’s syndrome patients according to vit D insufficiency.

	Vit D ≥ 30			
HRR	T1: <0.87	T2: 0.87–0.98	T3: >0.98	*p *-Value
N: 23	10	6	7	
TAPSE, mm	24.2 ± 4.58	22.16 ± 5.19	24.4 ± 4.01	>0.05
STDI, cm/s	11.97 ± 3.25	23.71 ± 5.33	12.98 ± 1.34	>0.05
PAPs, mmHg	29.80 ± 10.17	35.83 ± 7.43	23.43 ± 6.29	>0.05
TAPSEPAPs	0.88 ± 0.3	0.89 ± 0.41	1.11 ± 0.43	>0.05
FAC_RV, %	39.01 ± 4.81	36.18 ± 9.37	42.85 ± 10.57	>0.05
EF_RV	49.36 ± 7.78	46.28 ± 5.56	44.76 ± 5.88	>0.05
	Vit D < 30			
N: 38	12	14	12	
TAPSE, mm	21.97 ± 4.36	23.99 ± 3.32	13.82 ± 10.3	>0.05
STDI, cm/s	12.05 ± 2.91	12.43 ± 2.58	12.57 ± 1.69	>0.05
PAPs, mmHg	29.58 ± 7.59	30.07 ± 4.9	22.67 ± 7.66	**0.016**
TAPSEPAPs	0.79 ± 0.26	0.81 ± 0.17	1.18 ± 0.46	**0.004**
FAC_RV, %	38.73 ± 12.48	40.66 ± 11.08	43.02 ± 11.56	>0.05
EF_RV	49.64 ± 5.56	46.06 ± 9.02	50.29 ± 6.99	>0.05

Data presented as mean ± SD; Kruskal–Wallis H test has been performed. HRR: hemoglobin-to-RDW ratio, TAPSE: tricuspid annular plane systolic excursion, PAP: systolic pulmonary arterial pressure, STDI: tricuspid annular systolic velocity, TAPSE/PAPs: TAPSE to PAPs ratio, FAC: fractional area change, RV: right ventricle, EF: ejection fraction. The bold numbers indicate the meaning of the *p*-value.

## Data Availability

The original contributions presented in this study are included in the article. Further inquiries can be directed to the corresponding authors.
